# A role for spleen monocytes in post-ischemic brain inflammation and injury

**DOI:** 10.1186/1742-2094-7-92

**Published:** 2010-12-15

**Authors:** Yi Bao, Eunhee Kim, Sangram Bhosle, Heeral Mehta, Sunghee Cho

**Affiliations:** 1 Burke-Cornell Medical Research Institute, 785 Mamaroneck Avenue, White Plains, NY 10605, USA; 2 Department of Neurology and Neuroscience, Weill Cornell Medical College, New York, NY 10016, USA

## Abstract

Although infiltration of peripheral monocytes/macrophages is implicated in stroke pathology, *in vivo *data regarding the deployment of monocytes and their mobilization to the infarct area is scarce. Recent literature showed that mouse monocytes exhibit two distinct populations that represent pro-inflammatory (Ly-6C^hi^/CCR2+) and anti-inflammatory (Ly-6C^low^/CCR2-) subsets and that spleen is a major source for monocyte deployment upon injury. By reducing post-ischemic infection with antibacterial moxifloxacin (MFX) treatment, the present study investigates the effect of the treatment on Ly-6C and CCR2 expression in the spleen following ischemia and the extent to which the effect is associated with attenuation of post-ischemic inflammation and injury. Mice subjected to a middle cerebral artery occlusion (MCAO) showed a significant reduction in their spleen weights compared to sham animals. Compared to vehicle controls, splenocytes obtained from daily MFX-treated mice 7 days after ischemia exhibited significantly reduced mean Ly-6C expression within pro-inflammatory subsets, whereas the distribution of pro- and anti-inflammatory subsets was not different between the treatment groups. Additionally, MFX treatment significantly reduced CCR2 expression in the spleen tissue and in the post-ischemic brain and attenuated infarct size. The study suggests a potential contributing role of spleen monocytes in post-ischemic inflammation and injury. The influence of peripheral inflammatory status on the primary injury in the CNS further implies that the attenuation of post-stroke infection may be beneficial in mitigating stroke-induced brain injury.

## Findings

Ischemia-reperfusion causes inflammation that attracts monocyte/macrophage cells to infarct [1-3]. Monocytes are circulating antigen-presenting leukocytes that play an important role in inflammation, T-cell differentiation, phagocytosis, and innate immunity [4,5]. It has been shown that circulating and spleen monocytes are similar in their morphology, phagocytic capability, and gene expression profiles [6]. The study also identified the spleen as a monocyte reservoir and their numbers in the spleen are several folds higher than in circulation [6]. In addition, the number of monocytes that migrate to the infarct area after a myocardial infarction well exceeds the number in circulation under homeostatic conditions [4]. These studies suggest a potential role of the spleen in deploying monocytes upon cerebral ischemia.

Human and mouse monocytes exhibit distinct subsets that are reminiscent of macrophage phenotypes [5,7,8]. In mice, the subset that expresses a high level of the hematopoietic cell differentiation antigen Ly-6C (Ly-6C^hi^) also expresses the G-protein linked membrane protein, CCR2. The Ly-6C^hi^/CCR2+ monocyte subset is specifically recruited to an injury site by monocyte chemoattractant protein-1 (MCP-1), which is produced by the inflamed tissue, and become classically activated M1 macrophages. In contrast, the Ly-6C^low ^monocyte subset expresses CX_3_CR1, a receptor for the chemokine CX_3_CL1 (fractalkine), but is devoid of CCR2 expression. This anti-inflammatory Ly-6C^low^/CCR2-/CX_3_CR1+ subset is recruited to normal tissue and develops into resident M2 macrophages that function in host defense and repair after injury [9,10]. Recruitment of the pro-inflammatory Ly-6C^hi^/CCR2+ subset to inflammatory sites is believed to be CCR2-dependent, since monocytes from CCR2-null mice do not traffic as efficiently into a myocardial infarct as CCR2+ monocytes [6]. Furthermore, CCR2-null mice were protective against cerebral inflammation following ischemia [11], suggesting that CCR2 is a contributing factor for stroke-induced injury.

Studies suggest a potential influence of peripheral inflammatory status on primary injury. Fever and systemic infections are frequently observed conditions in patients suffering from stroke and are associated with increased mortality and poorer outcome [12,13]. Treatment with antibacterial agents such as moxifloxacin (MFX) and minocycline was shown to reduce infarct in experimental animal models of stroke [14,15]. In addition, MFX treatment also reduced peripheral infection in patients who have suffered an ischemic stroke and in animal models of stroke [16]. The present study investigates whether improving peripheral infection by treatment with MFX shifts spleen monocytes to a less pro-inflammatory state and if the effect is associated with attenuation of post-ischemic inflammation and injury. Here, we report a potential influence of peripheral inflammatory status on stroke-induced inflammation and injury.

All experimental procedures on animals were approved by the Institutional Animal Care and Use Committee of Weill Medical College of Cornell University. C57BL/6 male mice obtained from Jackson Laboratory (Bar Harbor, ME) were subjected to a 40 min middle cerebral artery occlusion (MCAO) as described previously [17,18]. The cerebral blood flow (CBF) in the center of the ischemic territory was monitored by laser-Doppler flowmetry (Periflux System 5010; Perimed, Jarfalla, Sweden). Moxifloxacin (MFX; Bayer, Wayne, NJ) solution (10 mg/ml) was prepared in a mixture of saline and 1 mol/L HCl (10:1) and adjusted to pH 7 with NaOH. Mice were treated with either vehicle or MFX solution (100 mg/kg) immediately after reperfusion, then once a day for 7 days following MCAO and sacrificed 7 days after ischemia. Sham-operated mice served as controls. Brains were removed, frozen, and sectioned (thickness, 20 μm) in a cryostat as previously described [17,18]. Brain sections were collected serially at 600 μm intervals, and stained with Cresyl Violet. Infarct volume was determined using Axiovision (Zeiss, Germany) and the contribution due to swelling was corrected.

The distribution of monocyte subsets and expression of Ly-6C were analyzed by flow cytometry/FACS according to a published method [6]. After removal of RBC using a lysis buffer (Sigma-Aldrich, St. Louis, MO), single splenocyte suspension was incubated with a cocktail of phycoerythrin (PE)-conjugated antibodies (BD Biosciences, San Jose, California) against T cells (CD90.2-PE, Clone 53-2.1), B cells (CD45R/B220-PE, Clone RA3-6B2), NK cells (CD49b/Pan-NK cells-PE, Clone DX5,; NK1.1-PE, Clone PK136), granulocytes (Ly-6G-PE, Clone 1A8), allophycocyanin (APC)-conjugated antibody against myeloid cells including monocytes/macrophages (CD11b-APC, Clone M1/70), and fluorescein isothiocyanate (FITC)-conjugated antibody to monocyte subsets (Ly-6C-FITC, Clone AL-21). After washing with Dulbecco's phosphate buffer saline (DPBS, VWR, West Chester, PA), 100,000 cells were analyzed by FACS. The selected gate (low PE/high APC which were identified as monocytes) was further analyzed for Ly-6C-FITC.

The procedures for RNA isolation and real time reverse transcriptase (RT)-PCR for gene expression were previously described [17]. Relative mRNA levels were quantified with real-time quantitative RT-PCR (qPCR) using fluorescent TaqMan technology. PCR primers specific for CCR2, MCP-1 and β-actin (internal control) were obtained as TaqMan pre-developed optimized assay reagents for gene expression (Applied Biosystems, Foster City, CA). The PCR reaction was performed using FastStart Universal Probe Master (Roche), according to the manufacturer's instructions. Reactions were performed in 20 μl total volumes and incubated at 95°C for 10 min, followed by 45 cycles of 15 s at 95°C and 1 min at 60°C. The results were analyzed by 7500 Fast Real-Time PCR System software. For CCR2 protein levels, spleen was homogenized in radioimmunoprecipitation assay buffer (RIPA buffer, Sigma-Aldrich) with freshly added protease inhibitors (Sigma-Aldrich) and centrifuged at 10,000 rpm for 10 min at 4°C. The protocol for SDS-PAGE and western blot were described previously [17]. After blocking the membrane for 1 h, anti-CCR2 monoclonal antibody (1:1000, ab32144, Abcam, Cambridge, MA) and anti-α-tubulin antibody as internal control (1:1000, T6199, Sigma-Aldrich) were added, followed by IRDye 680 Goat anti-rabbit IgG and IRDye 800 Donkey anti-mouse IgG for CCR2 and for α-tubulin respectively (1:10,000, LI-COR). The bands were visualized using the Odyssey Imaging System (LI-COR).

**Figure 1:**
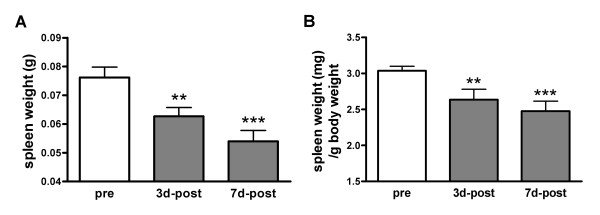
**Effect of ischemia on spleen weight**. A, Quantification of spleen weight before (pre) and after (post) ischemia. B, Quantification of spleen weight expressed ratios of spleen to body weight. Pre, control mice, n = 9; 3d-post, 3 days after MCAO, n = 6; 7d-post, 7 days after MCAO, n = 10. Values were expressed as mean ± SEM. ** *p *< 0.01 and *** *p *< 0.001 vs pre.

To investigate involvement of the spleen in stroke pathology, spleen weights were determined after MCAO. Ischemia caused significant reductions in spleen weight at 3 and 7 days post-ischemia with a larger reduction at 7 days (Figure 1A). Since body and spleen weights are significantly correlated in normal mice (R^2 ^= 0.85, p < 0.001, n = 13), we consider the possibility that the reduced spleen weights are due to body weight loss following stroke. Normalization of spleen weight by body weight still revealed significant weight reductions in the spleen (Figure 1B), indicating a potential involvement of spleen in stroke pathology.

**Figure 2:**
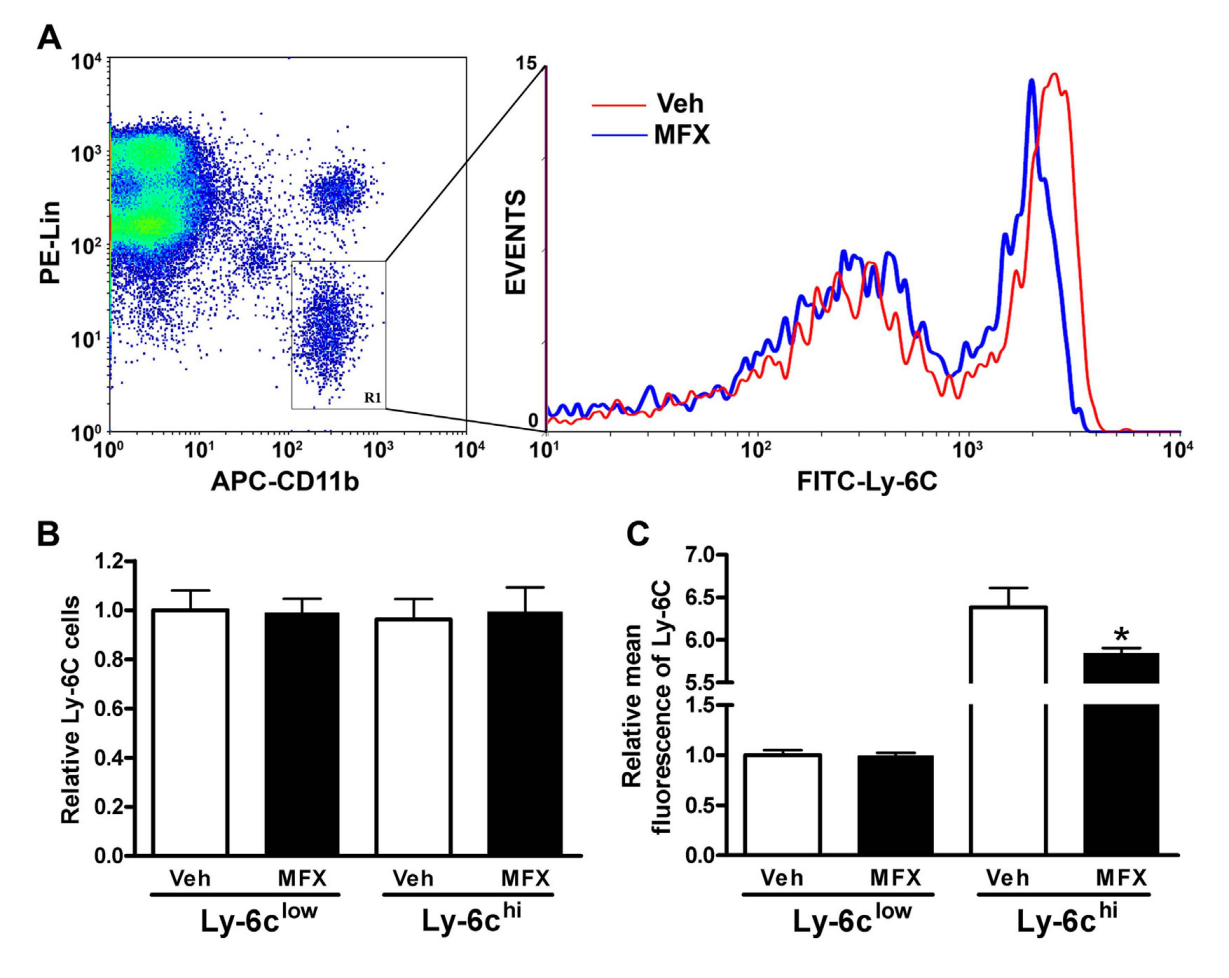
**Effect of MFX on monocyte subset distribution and Ly-6C expression in the spleen following stroke**. A, The monocyte subsets were identified by selecting the gate (APC-CD11b^hi ^and PE-Lin^low ^in left panel) and further separated by FITC-Ly-6C^hi/low ^(right panel). PE-Lin: PE-conjugated CD90/B220/CD49b/NK1.1/Ly-6G; APC-CD11b: APC-conjugated CD11b; FITC-Ly-6C: FITC-conjugated Ly-6C. B & C, Quantification of spleen monocyte subsets by their distribution (B) and mean Ly-6C intensities within each subset (C). n = 5/group. * *p *< 0.05 vs Veh.

**Figure 3:**
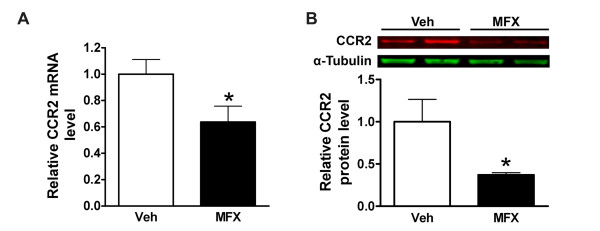
**Effect of MFX on CCR2 expression in the spleen following stroke**. CCR2 mRNA (A, n = 8/group) and protein (B, n = 5/group) levels were determined in the spleen at 7 days post-ischemia. Values were expressed as mean ± SEM. * *p *< 0.05 compared to vehicle (Veh).

Spleen monocytes were shown to resemble circulating monocytes in their morphology, phagocytic capability and transcriptome [6]. To reflect peripheral inflammatory state, we treated mice with vehicle or MFX after MCAO and analyzed the spleen weights and monocyte subsets in the spleen. Compared to pre-ischemic spleen weight (76.2 ± 3.6 mg, n = 9), stroke caused similar reductions in spleen weight between vehicle- and MFX-treated mice at 7 days post-ischemia (49.86 ± 7.92 mg vs 47.95 ± 5.66 mg, ns, n = 10-12). The lack of difference in weight at this time point may be due to immediate deployment of spleen monocytes upon injury as the spleen is an immediate source for monocytes [6]. Further FACS analyses in the selected gate for monocytes (Figure 2A, R1) revealed no differences in subset distribution between the treatment groups. However, MFX-treated mice showed reduction in mean Ly-6C expression within pro-inflammatory Ly-6C^hi ^subset (Figure 2). The reduced mean Ly-6C expression in MFX-treated mice was also associated with attenuated CCR2 gene and protein expression (Figure 3A &3B). The findings are consistent with other reports showing increased Ly-6C expression in response to pro-inflammatory stimuli [19,20] and reduced expression of cytokines and Ly-6C in CCR2-null mice [6,21,22]. Collectively, our findings indicate that MFX treatment following stroke shifts the spleen toward a less inflammatory state.

**Figure 4:**
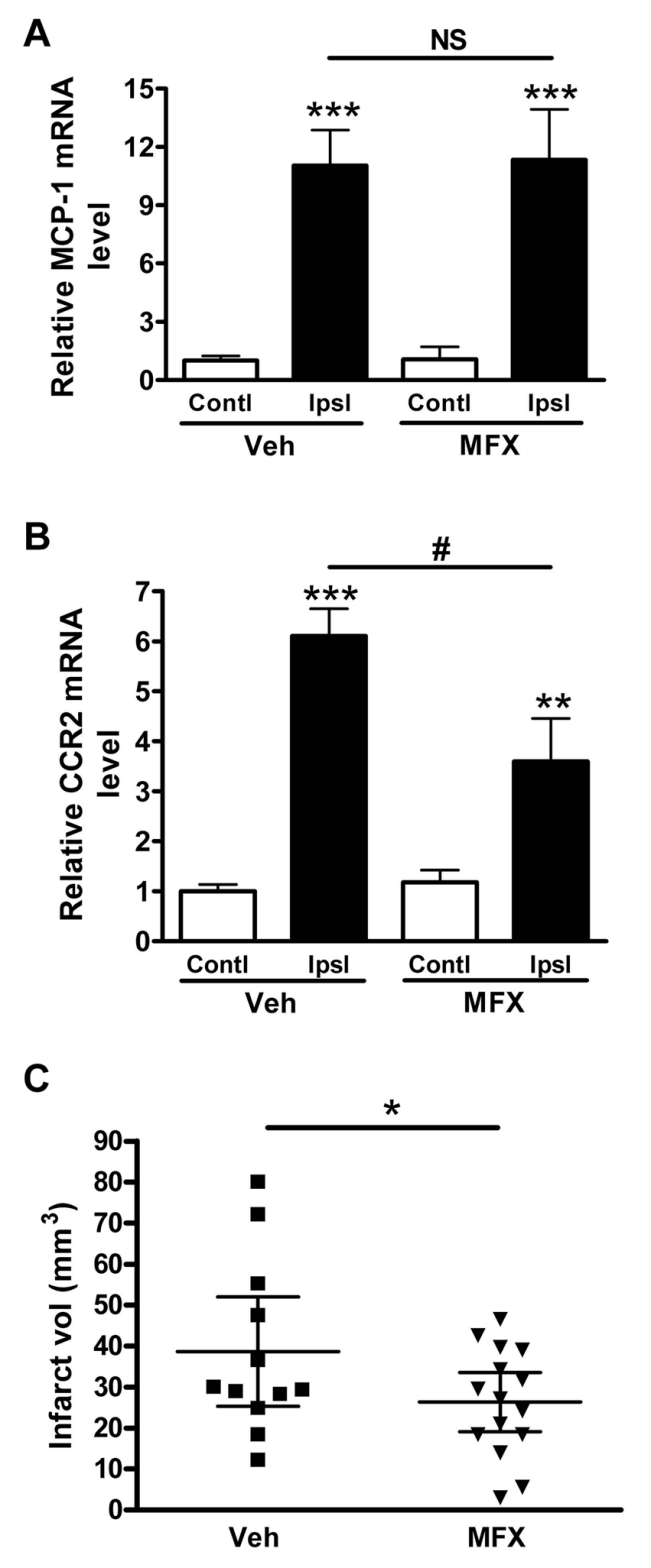
**Effect of MFX on post-ischemic inflammation and injury in the brain**. MCP-1 mRNA (A) and CCR2 mRNA (B) levels were measured in the 7 d post-ischemic brain in vehicle (Veh) and MFX treated mice. CCR2 mRNA level (B) was decreased by MFX, and MCP-1 was not significant between Veh and MFX treatment (A). β-actin was used as internal control and data were normalized with the contralateral side in Veh group. Values were expressed as mean ± SEM. Contl, contralateral side; Ipsl, ipsilateral side. ** *p *< 0.01 and *** *p *< 0.001 compared to contralateral side; # *p *< 0.05 compared to Veh; NS, not significant. n = 6-8/group. C, Infarct volume measurement 7 d after MCAO. Values were present as mean ± 95% Confidence interval. * *p *< 0.05 compared to Veh. n = 12-15/group.

We further investigated whether the MFX treatment influences the expression of MCP-1 and CCR2, a primary inflammatory axis and injury in the ischemic brain. Stroke increased MCP-1 mRNA levels in the infarct similarly in both groups (Figure 4A). Interestingly, MFX treated group showed a significant reduction in CCR2 mRNA levels in the post-ischemic brain, suggesting either less CCR2+ cell infiltration or reduced CCR2 expression in infiltrated cells to the injury site (Figure 4B). This was accompanied by a modest but significant reduction in infarct size (Figure 4C).

Despite a neurocentric view of stroke-induced brain injury, infiltration of peripheral monocytes/macrophages into the infarct territory indicates the influence of these immune cells in developing brain injury [23-26]. Although controversial, approximately 10-20% of infiltrated cells express markers for monocytes/macrophages [27,28]. With reported peripheral studies showing that prolonged systemic inflammation following stroke exacerbates brain injury and MFX treatment reduces the peripheral infection and improves ischemic outcome [14,16], the present study addresses the biological events that underlie the peripheral influence on stroke injury. Although recruited monocytes are thought to arise from systemic circulation and bone marrow, the unexpected finding that identifies the spleen as an immediate reservoir to deploy a large quantity of monocytes upon injury [6], argues for a novel role of spleen monocytes in stroke pathology.

The MFX-induced shift to less inflammatory status in the periphery was accompanied by reduced CCR2 expression in the post-ischemic brain (Figure 4B). CCR2 is a chemokine receptor for MCP-1, and the CCR2/MCP-1 axis plays a critical role in a host of inflammatory conditions [11,29,30]. Although it has been shown that other CNS cells, such as neurons and microglia, also express CCR2 in pathological conditions [31], it is believed that the major source of CCR2 in the post-ischemic brain is from the periphery. The reduced CCR2 expression, thereby attenuating post-ischemic inflammation in MFX-treated mice, may be due to reduced trafficking of monocytes to the injury site and/or attenuated CCR2 expression among recruited cells. Since we found that MFX treatment reduces Ly-6C expression in the pro-inflammatory subset (Figure 2), these findings support the view that a shift of spleen Ly-6C^hi ^subset to a less inflammatory phenotype may account for the reduced CCR2 expression in MFX-treated mice.

Consistent with literatures showing that MFX improves ischemic outcome and reduces peripheral infection both in animal models and patients [14,16,32,33], we also observed that MFX treatment resulted in a moderate but significant reduction in injury size. The MFX-treated mice also exhibited a smaller interanimal variability in injury size (Figure 4C). This raises an intriguing notion that the neuroprotection effects may not be caused by a primary ischemia-reperfusion event but by a secondary event such as reduced expression of inflammatory mediator in peripheral immune cells. The degree of ischemic severity was comparable between the treatment groups indicated by no noticeable differences in the degree of cerebral blood flow reduction during (MFX 83.8 ± 2.3% vs vehicle 83.6 ± 2.0%) and reperfusion 10 min after ischemia (MFX 122.6 ± 7.2% vs vehicle 134.7 ± 9.2%) and in MCP-1 mRNA levels produced in the post-ischemic brain (Figure 4A). MFX significantly decreased the WBC counts in circulation (2264 ± 259 vs 960 ± 60 cells/μl, n = 7-10, p < 0.01). Further FACS analysis in the spleen revealed the reduced percentage of monocyte population in the MFX-treated group (4.1 ± 1.0% vs 2.9 ± 0.4%, n = 5, p < 0.05). Since elevated systemic inflammation induces monocytosis, the decreased population of spleen monocytes by MFX treatment indicates attenuation of peripheral inflammation following stroke. Therefore, the protective mechanism may lie in limiting the expansion of secondary injury associated with the reduced inflammatory state in monocytes.

In summary, antibacterial MFX treatment after stroke shifted inflammatory status in the spleen toward a less inflammatory state and attenuated post-ischemic inflammation and injury. The tight association between MFX-induced neuroprotection and inflammatory status in the brain and spleen suggests that reducing peripheral inflammation may be beneficial in ameliorating post-ischemic inflammation and injury. The potential influence of inflammatory status in peripheral monocytes on CNS injury suggests a possible cell-based strategy to limit the expansion of secondary injuries during post-ischemic periods.

## Competing interests

The authors declare that they have no competing interests.

## Authors' contributions

YB contributed to design of the study, performed the western blot, Real-Time PCR and FACS analysis, reviewed and organized the data and wrote the manuscript; EK contributed to developing the concept of the involvement of spleen in stroke pathology, the spleen weight measurement and reviewed the manuscript; SB performed the MCAO surgery, white blood cell counting and reviewed the manuscript; HM performed tissue preparation and infarct analysis and reviewed the manuscript; SC directed the overall study, analysis of the data, and reviewed the manuscript. All authors have read and approved the final manuscript.
